# Predictors of mortality of *Staphylococcus aureus* bacteremia among patients hospitalized in a Swiss University Hospital and the role of early source control; a retrospective cohort study

**DOI:** 10.1007/s10096-023-04557-1

**Published:** 2023-02-02

**Authors:** Matthaios Papadimitriou-Olivgeris, Giorgia Caruana, Laurence Senn, Benoit Guery

**Affiliations:** 1 Infectious Diseases Service, Lausanne University Hospital, Lausanne, Switzerland; 2 Infection Prevention and Control Unit, Lausanne University Hospital, Lausanne, Switzerland; 3 Institute of Microbiology, Lausanne University Hospital, Lausanne, Switzerland

**Keywords:** Methicillin-resistant *Staphylococcus aureus* (MRSA), Source control, Infective endocarditis, Infectious diseases consultation, Sepsis, Bloodstream infection

## Abstract

*S. aureus* bacteremia is associated with high mortality. The aim was to identify predictors of mortality among patients with *S. aureus* bacteremia and evaluate the role of early source control. This retrospective study was conducted at the Lausanne University Hospital, Switzerland. All episodes of *S. aureus* bacteremia among adult patients from 2015 to 2021 were included. During the study period, 839 episodes of *S. aureus* bacteremia were included, of which 7.9% were due to methicillin-resistant isolates. Bacteremias were related to bone or joint infections (268; 31.9%), followed by bacteremia of unknown origin (158; 18.8%), proven endocarditis (118; 14.1%) and lower-respiratory tract infections (79; 9.4%). Overall 28-day mortality was 14.5%. Cox multivariate regression model showed that Charlson comorbidity index > 5 (*P* < 0.001), nosocomial bacteremia (*P* 0.019), time to blood culture positivity ≤ 13 h (*P* 0.004), persistent bacteremia for ≥ 48 h (*P* 0.004), sepsis (*P* < 0.001), bacteremia of unknown origin (*P* 0.036) and lower respiratory tract infection (*P* < 0.001) were associated with 28-day mortality, while infectious diseases consultation within 48 h from infection onset (*P* < 0.001) was associated with better survival. Source control was warranted in 575 episodes and performed in 345 episodes (60.0%) within 48 h from infection onset. Results from a second multivariate analysis confirmed that early source control (*P* < 0.001) was associated with better survival. Mortality among patients with *S. aureus* bacteremia was high and early source control was a key determinant of outcome. Infectious diseases consultation within 48 h played an important role in reducing mortality.

## Introduction

*Staphylococcus aureus* is one of the most common causes of community and hospital-acquired bacteremias [1]. Due to its complexity, a holistic approach incorporating diagnostic workup (follow-up blood cultures, echocardiography, metastatic foci identification) and management (antimicrobial treatment and source control) is needed to improve outcome [2–4]. Despite such an approach, mortality remains high, ranging from 21 to 42% [5–10].

Several factors have been associated with worst outcome among patients with *S. aureus* bacteremia, such as age, comorbidities [5, 6, 9–11], presence of sepsis or septic shock [6, 9, 12, 13], immunosuppression [14, 15], and specific foci of infection, such as pneumonia, endocarditis or bacteremia of unknown origin [5, 8, 9, 11]. Although, aforementioned factors are unmodifiable, management of bacteremia can also impact outcome; appropriate antimicrobial treatment was repeatedly shown to improve outcome [6, 7, 16]. Source control is also a key step in early management of infected patients; however, controversy exists concerning the rapidity of source control achievement, with some studies showing an improved survival [6, 7], while in others early source control did not confer significant survival benefit [2, 16].

The aim of the present study was to identify predictors of mortality in patients with *S. aureus* bacteremia and evaluate the role of source control in a Swiss tertiary university hospital.

## Materials and methods

We conducted a retrospective study at the Lausanne University Hospital, Switzerland during a seven-year period (2015–2021). The Lausanne University Hospital is a 1100-bed primary and tertiary care hospital with 35 intensive care units (ICU) beds. The study was approved by the ethic committee of the Canton of Vaud (CER-VD 2021–02,516) that waived the need for informed consent allowing the inclusion of all hospitalized patients except those who refused the use of their clinical and laboratory data.

Inclusion criteria were adult patients (≥ 18 years old) and presence of at least one blood culture for *S. aureus* (database of the microbiology laboratory). Exclusion criteria were patients’ written refusal of the use of their data and incomplete medical files (patients transferred to other hospital upon infection onset without follow-up information).

Blood cultures were incubated the BacT/ALERT System (bioMerieux, Marcy l'Etoile, France). Matrix-assisted laser desorption-ionization time of flight mass spectrometry (MALDI-TOF MS; Bruker Daltonics, Bremen, Germany) was used for the identification to the species level. Susceptibility results were collected from the microbiology laboratory database and evaluated according the EUCAST criteria [17].

Twenty-eight-day mortality was the primary outcome. Data regarding demographics (age, sex), comorbidities, Charlson Comorbidity Index [18], laboratory results (white blood cells, platelets, C-reactive protein, procalcitonin) on the day of first positive blood culture, Sequential Organ Failure Assessment (SOFA) score [19], antimicrobial treatment, source control, presence of sepsis or septic shock, infection site were retrieved from patients’ electronic health records. All data were collected, stored and managed using REDCap by an infectious diseases specialist. REDCap electronic data capture tools is hosted at Lausanne University Hospital. REDCap (Research Electronic Data Capture) is a secure, web-based software platform designed to support data capture for research studies [20, 21].

The date of collection of the first positive blood culture was defined as infection onset. A new episode was included if more than 30 days had elapsed since the first positive blood culture. Since 2007, an infectious diseases consultation was performed on a mandatory basis within the same day of *S. aureus* blood culture positivity [3].

Bacteremia was characterized as community if the first positive blood culture was drawn upon hospital admission or within 48 h after hospital admission and nosocomial if the first positive blood cultures were drawn after 48 h from hospital admission. Sepsis or septic shock was defined according to definition proposed by the Sepsis-3 International Consensus [22]. Complicated bacteremia was defined as presence of endocarditis, metastatic infection, implanted prostheses or persistent bacteremia for more than 48 h. Infectious endocarditis was defined according to the modified Duke criteria [23]. Cardiac predisposing factors for endocarditis were defined as cardiac conditions at high or moderate risk for infectious endocarditis [24]. Infection site was defined by the infectious diseases consultant responsible of the case on the basis of clinical, radiological, microbiological, and operative findings. Appropriate antimicrobial treatment was defined as one that included an antimicrobial agent with in vitro activity against the infecting isolate, initiated within 24 h from the infection onset, at an adequate dosage. Source control considered as warranted was (1) removal of venous catheter in patients with bacteremia of unknown origin in the presence of vascular catheter or catheter-related bacteremia; (2) surgical or imaging-guided drainage of infected collections (abscess, peritonitis, and empyema); (3) joint fluid drainage (arthrotomy or arthroscopy); (4) cardiac surgery in endocarditis patients when indicated [23]; (5) correction of urinary-tract obstruction. Early source control was defined if performed within 48 h from infection onset.

SPSS version 26.0 (SPSS, Chicago, IL, USA) and R version 4.1.3 (2022, Vienna, Austria) statistical soft wares were used for data analysis. Categorical variables were analyzed using the chi-square or Fisher exact test and continuous variables with Mann–Whitney *U* test. Univariate logistic regression models were assessed with 28-mortality as dependent variable. Covariates were tested for multi-collinearity through variance inflation factor assessment: those not collinear and clinically relevant were used in multivariate analysis. After checking Cox assumptions, two multivariate Cox proportional hazards regression models were performed with 28-day mortality as the time-to-event: (i) first including all patients, (ii) second assessing only patients for whom a source control was needed based on the type of the infection. Odds ratios (ORs) and 95% confidence intervals (CIs) were calculated to evaluate the strength of any association. All statistic tests were 2-tailed and *P* < 0.05 was considered statistically significant. We finally performed Kaplan–Meier curves of the survival probability of patients with *S. aureus* bacteremia according to appropriate source control with 48 h from infection onset and presence of septic shock. Since it was previously suggested that source control could be influenced by care withdrawal [25], Kaplan–Meier curve was performed among patients that were alive and in maximal care for 7 days after infection onset in order to assess the role of early source control on survival.

## Results

**Fig. 1:**
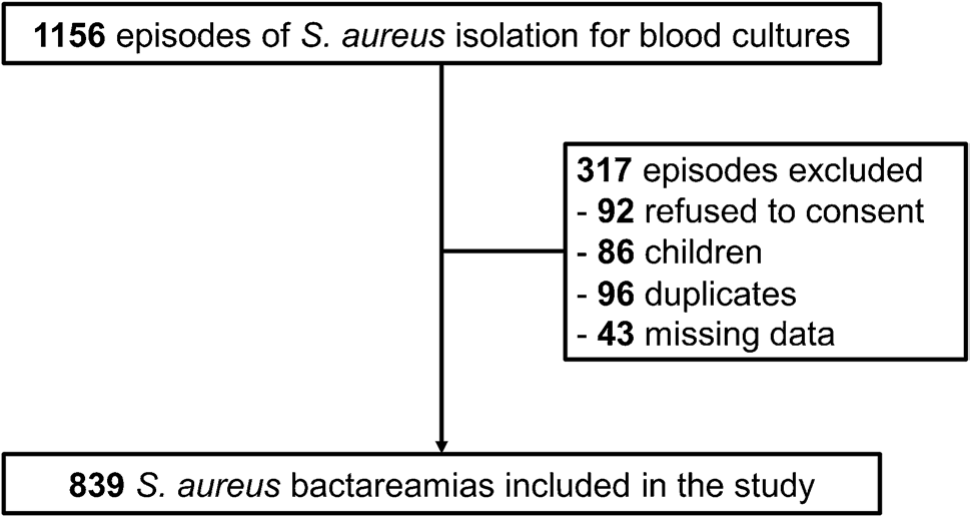
Flowchart of patients’ selection

A total of 1156 episodes of *S. aureus* bacteremia were identified; 839 episodes in 779 patients were included (Fig. 1). Forty-seven patients had multiple episodes (41, 5, and 1 patients had 2, 3, and 4 episodes, respectively). The 60 subsequent episodes of bacteremia, occurred at a median of 8 months from the previous episode (range 1–73 months). Overall, 66 (7.9%) isolates were resistant to methicillin. Seventy-seven (9.2%) episodes were polymicrobial. Most bacteremias were related to bone and joint infections (268; 31.9%), followed by bacteremia of unknown origin (158; 18.8%), proven endocarditis (118; 14.1%), lower-respiratory tract (79; 9.4%), and central catheter (78; 9.3%). Among episodes with endocarditis, 105 had valvular infection (cardiac surgery in 36 patients among 56 with indication) and 21 lead infections of cardiovascular implantable electronic devices (CIEDs; CIED removal in 20 patients).

Overall 28-day mortality rate was 14.5% (122 episodes). Results of univariate analysis for predictors of 28-day mortality are shown in Table 1. Sepsis occurred in 352 (42.0%) episodes. Antimicrobial treatment was initiated within 24 h in 801 (95.5%) episodes and was appropriate in 761 (90.7%) episodes. Infectious diseases consultation was provided in 727 (86.7%) cases within 48 h from infection onset. Results from Cox multivariate regression model showed that Charlson comorbidity index > 5 (*P* < 0.001; OR 4.98, CI 2.61–9.48), nosocomial bacteremia (*P* 0.019; OR 1.57, CI 1.08–2.29), time to blood culture positivity ≤ 13 h (*P* 0.004; OR 1.85, CI 1.22–2.81), persistent bacteremia for ≥ 48 h (*P* 0.004; OR 1.83, CI 1.22–2.76), sepsis (*P* < 0.001; OR 3.39, CI 1.97–5.83), bacteremia of unknown origin (*P* 0.036; OR 1.64, CI 1.03–2.60) and lower respiratory tract infection (*P* < 0.001; OR 2.96, CI 1.77–4.95) were associated with 28-day mortality, while infectious diseases consultation within 48 h from infection onset (*P* < 0.001; OR 0.45, CI 0.30–0.69) was associated with better survival.

**Table 1:** Predictors of 28-day mortality of *S. aureus* bacteremia episodes

	Univariate analysis							Cox PH multivariate regression	
	Total (*n* = 839)		Survivors (*n* = 717)		Non-survivors (*n* = 122)		*P*	*P*	OR (95% CI)
Demographics									
Male sex	598	71.3%	513	71.5%	85	69.7%	0.672		
Age (years)	68	55–78	66	53–77	76	69–82	< 0.001		
Age > 60 years	553	65.9%	447	62.3%	106	86.9%	< 0.001	0.692	1.14 (0.60–2.15)
Co-morbidities									
Diabetes mellitus	251	29.9%	213	29.7%	38	31.1%	0.748		
Obesity (body mass index ≥ 30 kg/m^2^)	213	25.4%	190	26.5%	23	18.9%	0.073		
Chronic kidney disease (moderate or severe)	196	23.4%	160	22.3%	36	29.5%	0.083		
Malignancy (solid organ or hematologic)	161	19.2%	122	17.0%	39	32.0%	< 0.001		
Immunosuppression	151	18.0%	125	17.4%	26	21.3%	0.303		
Chronic obstructive pulmonary disease	96	11.4%	79	11.0%	17	13.9%	0.350		
Cirrhosis	76	9.1%	61	8.5%	15	12.3%	0.178		
Congestive heart failure	59	7.0%	41	5.7%	18	14.8%	< 0.001		
Charlson Comorbidity Index	5	3–8	5	2–7	8	6–9	< 0.001		
Charlson Comorbidity Index > 5	390	46.5%	297	41.4%	93	76.2%	< 0.001	< 0.001	4.98 (2.61–9.48)
Setting of infection onset									
Community	536	63.9%	470	65.6%	66	54.1%			
Nosocomial	303	36.1%	247	34.4%	56	45.9%	0.015	0.019	1.57 (1.08–2.29)
Cardiac predisposing factors	137	16.3%	121	16.9%	16	13.1%	0.299		
Presence of prosthetic material (other than cardiac valve)									
Bone or joint prosthetic material	191	22.8%	164	22.9%	27	22.1%	0.857		
CIED	88	10.5%	69	9.6%	19	15.6%	0.047		
Endovascular (non-cardiac) prosthetic material	48	5.7%	39	5.4%	9	7.4%	0.394		
Microbiological data									
Two or more blood cultures positive (initial blood cultures)	652	77.7%	551	76.8%	101	82.8%	0.145		
Polymicrobial bacteremia^a^	77	9.2%	67	9.3%	10	8.2%	0.685		
Methicillin-resistance	66	7.9%	52	7.3%	14	11.5%	0.109		
Urine culture positive for *S. aureus* (*n* = 466 patients)	66	7.9%	69	17.3%	10	14.7%	0.593		
Prior *S. aureus* bacteremia^b^	75	8.9%	62	8.6%	13	10.7%	0.472		
Time to blood culture positivity (h)	13	10–17	13	10–17	12	9–15	0.015		
Time to blood culture positivity ≤ 13 h	455	54.2%	372	51.9%	83	68.0%	0.001	0.004	1.85 (1.22–2.81)
Duration of bacteremia (h)	0	0–48	0	0–44	22	0–66	0.020		
Persistent bacteremia (≥ 48 h)	207	24.7%	162	22.6%	45	36.9%	0.001	0.004	1.83 (1.22–2.76)
SARS-CoV-2 infection	35	4.2%	25	3.5%	10	8.2%	0.016		
Infection data									
Fever	702	83.7%	606	84.5%	96	78.7%	0.107		
Infectious diseases consultation	786	93.7%	689	96.1%	97	79.5%	< 0.001		
Infectious diseases consultation within 48 h from infection onset	727	86.7%	636	88.7%	91	74.6%	< 0.001	< 0.001	0.45 (0.30–0.69)
Heart murmur	260	31.0%	219	30.5%	41	33.6%	0.499		
Embolic events	132	15.7%	106	14.8%	26	21.3%	0.067	0.064	1.58 (0.97–2.56)
Trunk	91	10.8%	79	11.0%	12	9.8%	0.698		
Cerebral	64	7.6%	46	6.4%	18	14.8%	0.001		
Limbs	30	3.6%	26	3.6%	4	3.3%	1.000		
Sepsis	352	42.0%	255	35.6%	97	79.5%	< 0.001	< 0.001	3.39 (1.97–5.83)
Septic shock	132	15.7%	86	12.0%	46	37.7%	< 0.001		
SOFA score	2	1–5	2	1–4	6	3–8	< 0.001		
SOFA score > 3	311	37.1%	223	31.1%	88	72.1%	< 0.001	0.074	1.71 (0.95–3.07)
Infection site									
Unknown origin	158	18.8%	127	17.7%	31	25.4%	0.044	0.036	1.64 (1.03–2.60)
Bone or joint	268	31.9%	246	34.4%	22	18.0%	< 0.001		
Proven endocarditis (including CIED cable infection)	118	14.1%	95	13.2%	23	18.9%	0.100		
Lower-respiratory tract	79	9.4%	55	7.7%	24	19.7%	< 0.001	< 0.001	2.96 (1.77–4.95)
Central venous catheter-related	78	9.3%	69	9.6%	9	7.4%	0.503		
Skin and soft tissue	66	7.9%	61	8.5%	5	4.1%	0.103		
Peripheral venous catheter-related	34	4.1%	29	4.0%	5	4.1%	1.000		
Other^c^	94	11.2%	87	12.1%	7	5.7%	0.038		
Complicated bacteremia	513	61.1%	435	60.7%	78	63.9%	0.494		
Laboratory data within 24 h from first positive blood culture									
White blood cells (× 10^9^/l)	12	9–17	12	9–17	12	7–16	0.323		
Neutropenia	26	3.1%	21	2.9%	5	4.1%	0.568		
Platelets (× 10^9^/l)	211	134–303	217	144–310	162	87–251	< 0.001		
C-reactive protein (mg/l) (= 778 patients)	202	103–304	180	103–293	220	116–306	0.014		
Procalcitonin (μg/l) (*n* = 149 patients)	2.6	0.5–15.2	2.1	0.5–12.5	8.7	2.5–28.6	0.001		
Treatment									
Empiric antimicrobial initiation (within 3 h)	656	78.2%	556	77.5%	100	82.0%	0.274		
Appropriate empiric antimicrobial (within 3 h)	625	74.5%	531	74.1%	94	77.0%	0.484		
Antimicrobial initiation within 24 h	801	95.5%	686	95.7%	115	94.3%	0.487		
Appropriate antimicrobial within 24 h	761	90.7%	654	91.2%	107	87.7%	0.217		
Source control (*n* = 575 patients)	533	92.7%	490	97.2%	43	60.6%	< 0.001		
Early source control (*n* = 575 patients)	345	60.0%	318	63.1%	27	38.0%	< 0.001		

*CIED* cardiac implantable electronic devices, *PH* proportional hazard, *SOFA* Sequential Organ Failure Assessment

Data are depicted as number and percentage or median and Q1–3

^a^32 streptococci, 26 Enterobacterales, 16 enterococci, 4 coagulase negative staphylococci, 4 *Pseudomonas aeruginosa*, 10 other species

^b^Prior episode occurred at a median of 10 months before the included episode (range 1–131 months)

^c^39 vascular infection (not-related to intravascular catheters), 23 intra-abdominal, 19 urinary-tract, 10 parotiditis, 3 central nervous system

Source control was warranted in 575 (68.5%) episodes and performed in 533 (92.7%); early source control was performed in 345 (60.0%) episodes. Table 2 shows the source control procedures warranted and those performed depending on infection site. Among the 575 episodes, 28-day mortality was 12.3%. Results from a second Cox multivariate regression model (Table 3) confirmed that Charlson comorbidity index > 5 (*P* < 0.001; OR 5.55, CI 2.65–11.62), nosocomial bacteremia (*P* < 0.001; OR 3.00, CI 1.75–5.14) and sepsis (*P* < 0.001; OR 5.46, CI 3.06–9.71) were associated with increased 28-day mortality, while infectious diseases consultation within 48 h from infection onset (*P* 0.002; OR 0.39, CI 0.22–0.71) and early source control (*P* < 0.001; OR 0.35, CI 0.20–0.60) were associated with better survival.

**Table 2:** Source control procedures warranted and performed depending on infection site

Infection site	Source control warranted	Type of source control procedure	Source control performed	Source control performed within 48 h from infection onset
Unknown origin (*n* = 158)	91 (57.6%)	Removal of central or peripheral venous catheter	86 (94.5%)	75 (82.4%)
Central venous catheter-related (*n* = 78)	78 (100%)	Removal of central venous catheter	76 (97.4%)	58 (74.4%)
Peripheral venous catheter-related (*n* = 34)	34 (100%)	Removal of peripheral venous catheter	34 (100%)	33 (97.1%)
Lower-respiratory tract (*n* = 79)	17 (21.5%)	Drainage of empyema	15 (88.2%%)	7 (41.2%)
Skin and soft tissue (*n* = 66)	33 (50.0%)	Drainage of abscess	32 (97.0%)	22 (66.7%)
Bone or joint (*n* = 268)	218 (81.3%)	Drainage of joint fluid or abscess, drainage or replacement of osteoarticular prosthetic material	201 (92.2%)	103 (47.2%)
Proven endocarditis^a^ (*n* = 118)	82 (69.5%)	Valvular replacement or removal of CIED	71 (86.6%)	14 (17.1%)
Other^b^ (*n* = 94)	60 (63.8%)	Replacement of vascular prothesis, treatment of mycotic aneurysm, drainage of intra-abdominal abscess, correction of urinary-tract, or biliary-tract obstruction	58 (96.7%)	33 (55.0%)

Data are depicted as number (percentage)

^a^Including CIED cable infection

^b^39 vascular infection (not-related to intravascular catheters), 23 intra-abdominal, 19 urinary-tract, 10 parotiditis, 3 central nervous system

**Table 3:** Predictors of 28-day mortality of *S. aureus* bacteremia in 575 episodes for which source control was indicated

		Univariate analysis						Cox PH multivariate regression	
	Total (*n* = 575)		Survivors (*n* = 504)		Non-survivors (*n* = 71)		*P*	*P*	OR (95% CI)
Demographics									
Male sex	404	70.3%	353	70.0%	51	71.8%	0.757		
Age (years)	66	55–77	65	54–76	75	69–82	< 0.001		
Age > 60 years	369	64.2%	307	60.9%	62	87.3%	< 0.001		
Co-morbidities									
Diabetes mellitus	168	29.2%	145	28.8%	23	32.4%	0.530		
Obesity (body mass index ≥ 30 kg/m^2^)	168	29.2%	154	30.6%	14	19.7%	0.060		
Chronic kidney disease (moderate or severe)	135	23.5%	111	22.0%	24	33.8%	0.028		
Malignancy (solid organ or hematologic)	107	18.6%	86	17.1%	21	29.6%	0.015		
Immunosuppression	102	17.7%	85	16.9%	17	23.9%	0.114		
Chronic obstructive pulmonary disease	56	9.7%	48	9.5%	8	11.3%	0.643		
Congestive heart failure	45	7.8%	37	7.3%	8	11.3%	0.249		
Cirrhosis	44	7.7%	35	6.9%	9	12.7%	0.089		
Charlson Comorbidity Index	5	3–7	5	2–7	7	6–9	< 0.001		
Charlson Comorbidity Index > 5	247	43.0%	193	38.3%	54	76.1%	< 0.001	< 0.001	5.55 (2.65–11.62)
Setting of infection onset									
Community	344	59.8%	309	61.3%	35	49.3%			
Nosocomial	231	40.2%	195	38.7%	36	50.7%	0.053	< 0.001	3.00 (1.75–5.14)
Cardiac predisposing factors	84	14.6%	74	14.7%	10	14.1%	0.894		
Presence of prosthetic material (other than cardiac valve)									
Bone or joint prosthetic material	138	24.0%	123	24.4%	15	21.1%	0.545		
CIED	64	11.1%	52	10.3%	12	16.9%	0.099		
Endovascular (non-cardiac) prosthetic material	40	7.0%	32	6.3%	8	11.3%	0.127		
Microbiological data									
Two or more blood cultures positive (initial blood cultures)	465	80.9%	405	80.4%	60	84.5%	0.405		
Polymicrobial bacteremia^a^	44	7.7%	38	7.5%	6	8.5%	0.811		
Methicillin-resistance	46	8.0%	36	7.1%	10	14.1%	0.044	0.136	1.68 (0.85–3.35)
Urine culture positive for *S. aureus* (*n* = 466 patients)	52	17.1%	43	16.2%	9	23.1%	0.289		
Prior *S. aureus* bacteremia^b^	48	8.3%	39	7.7%	9	12.7%	0.159		
Time to blood culture positivity (h)	13	10–17	13	10–17	12	9–16	0.368		
Time to blood culture positivity ≤ 13 h	313	54.4%	265	52.6%	48	67.6%	0.017	0.077	1.57 (0.95–2.60)
Duration of bacteremia (h)	0	0–50	0	0–46	32	0–82	0.010		
Persistent bacteremia (≥ 48 h)	146	25.4%	118	23.4%	28	39.4%	0.004		
SARS-CoV-2 infection	21	3.7%	16	3.2%	5	7.0%	0.164		
Infection data									
Fever	496	86.3%	438	86.9%	58	81.7%	0.267		
Infectious diseases consultation	552	96.0%	491	97.4%	61	85.9%	< 0.001		
Infectious diseases consultation within 48 h from infection onset	512	89.0%	456	90.5%	56	78.9%	0.003	0.002	0.39 (0.22–0.71)
Heart murmur	174	30.3%	148	29.4%	26	36.6%	0.213		
Embolic events	96	16.7%	81	16.1%	15	21.1%	0.307		
Limbs	26	4.5%	23	4.8%	3	4.2%	1.000		
Trunk	63	11.0%	57	11.3%	6	8.5%	0.470		
Cerebral	45	7.8%	35	6.9%	10	14.1%	0.036		
Sepsis	223	38.8%	167	33.1%	56	78.9%	< 0.001	< 0.001	5.46 (3.06–9.71)
Septic shock	80	13.9%	58	11.5%	22	31.0%	< 0.001		
SOFA score	2	1–4	2	1–4	6	3–8	< 0.001		
SOFA score > 3	196	34.1%	146	29.0%	50	70.4%	< 0.001		
Infection site									
Unknown origin	91	15.8%	72	14.3%	19	26.8%	0.007		
Bone or joint	218	37.9%	200	39.7%	18	25.4%	0.020		
Proven endocarditis (including CIED cable infection)	82	14.3%	67	13.3%	15	21.1%	0.077		
Central venous catheter-related	78	13.6%	69	13.7%	9	12.7%	0.815		
Peripheral venous catheter-related	34	5.9%	29	5.8%	5	7.0%	0.596		
Skin and soft tissue	33	5.7%	32	6.3%	1	1.4%	0.106		
Lower-respiratory tract	17	3.0%	16	3.2%	1	1.4%	0.709		
Other^c^	60	10.4%	56	11.1%	4	5.6%	0.212		
Complicated bacteremia	372	64.7%	320	63.5%	52	73.2%	0.108		
Laboratory data within 24 h from first positive blood culture									
White blood cells (× 10^9^/l)	13	9–17	13	9–17	12	8–16	0.288		
Neutropenia	18	3.1%	15	3.0%	3	4.2%	0.477		
Platelets (× 10^9^/l)	216	141–312	220	146–314	144	84–248	< 0.001		
C-reactive protein (mg/l) (*n* = 575 patients)	213	113–315	201	118–310	214	170–297	0.407		
Procalcitonin (μg/l) (*n* = 102 patients)	2.4	0.5–9.3	2.1	0.4–7.2	7.0	1.9–10.7	0.061		
Treatment									
Empiric antimicrobial initiation (within 3 h)	445	77.4%	386	76.6%	59	83.1%	0.288		
Appropriate empiric antimicrobial (within 3 h)	422	73.4%	369	73.2%	53	74.6%	0.886		
Antimicrobial initiation within 24 h	548	95.3%	480	95.2%	68	95.8%	0.841		
Appropriate antimicrobial within 24 h	518	90.1%	457	90.7%	61	85.9%	0.206		
Source control	533	92.7%	490	97.2%	43	60.6%	< 0.001		
Early source control	345	60.0%	318	63.1%	27	38.0%	< 0.001	< 0.001	0.35 (0.20–0.60)

*CIED* Cardiac implantable electronic devices, *PH* proportional hazard, *SOFA* Sequential Organ Failure Assessment

Data are depicted as number and percentage or median and Q1–3

^a^19 Enterobacterales, 16 streptococci, 12 enterococci, 4 coagulase negative staphylococci, 3 *Pseudomonas aeruginosa*, 8 other species

^b^Prior episode occurred at a median of 8 months before the included episode (range 2–131 months)

^c^27 vascular infection (not-related to intravascular catheters), 16 intra-abdominal, 14 urinary-tract, 2 parotiditis, 1 central nervous system

**Fig. 2:**
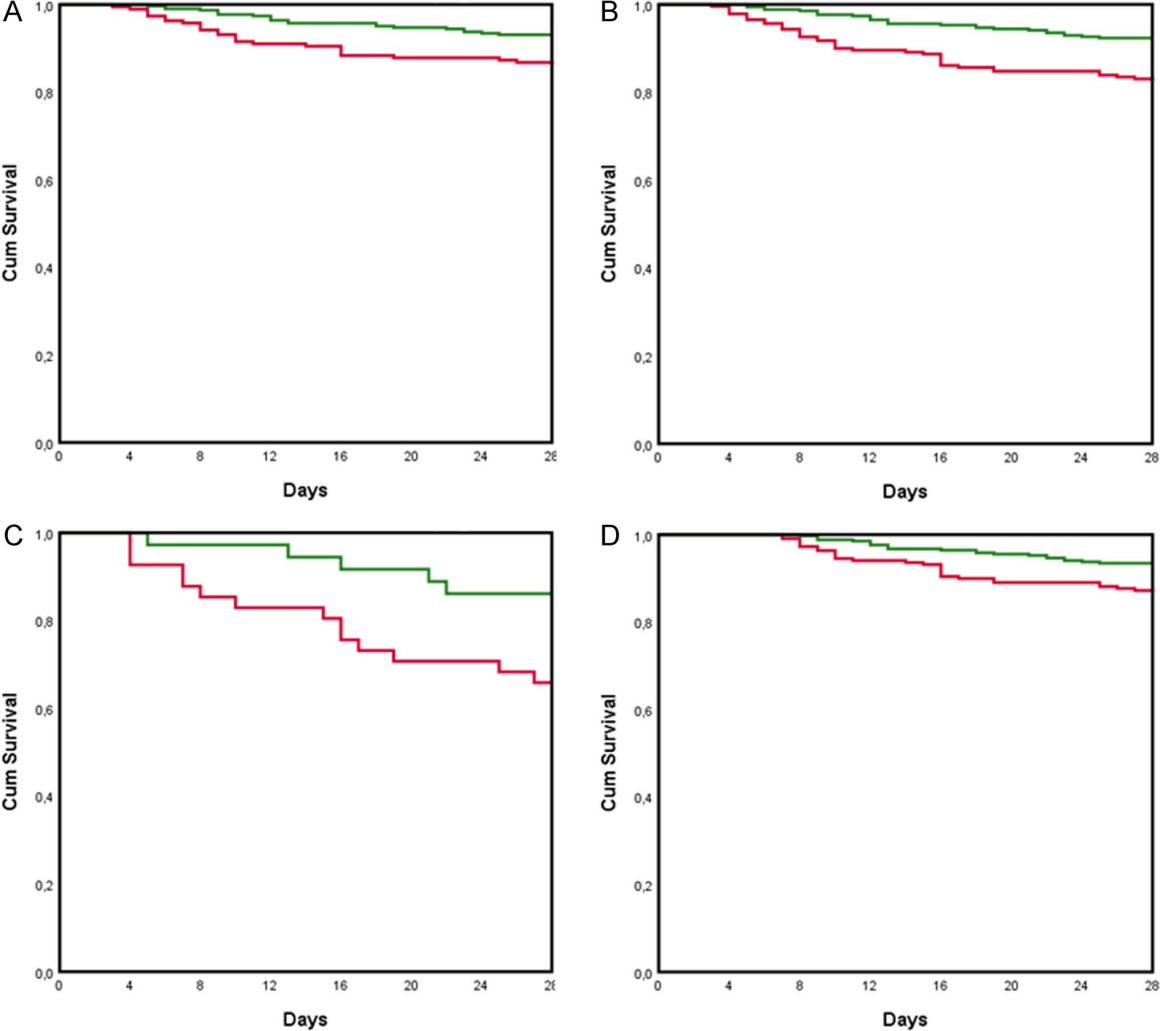
Kaplan–Meier curves of the survival probability of patients with *S. aureus* according to early source control **A** in 575 episodes for which source control was warranted, **B** in episodes without septic shock, **C** with septic shock. **D** The Kaplan–Meier curve among 555 patients that were alive and in maximal care for 7 days after infection onset. Red line: no early source control, green line: early source control

Figure 2 shows Kaplan–Meier curves of the survival probability of episodes with *S. aureus* according to early source control (A) in 575 episodes for which source control was warranted, (B) in episodes without septic shock, (C) with septic shock. Early source control was associated with better outcome in all episodes (*P* < 0.001) and in the subgroups of patients without (*P* 0.009) and with septic shock (*P* 0.035). Figure 2D shows the Kaplan–Meier curve among 555 episodes in patients that were alive and in maximal care for 7 days after infection onset; early source control was associated with better outcome (*P* 0.007).

## Discussion

The present study assessing factors associated with mortality among patients with *S. aureus* bacteremia highlights the crucial role of early interventions, such as source control and infectious diseases consultation on the management of such bacteremic patients.

The reported 28-day mortality rate was 14.5% which is lower than that reported in the literature (21–42%) [5–10]. One hypothesis of the increased mortality among aforementioned studies could be due to the higher rate of MRSA as compared to the present study (7.9%) [6–10]; the rate of MRSA in the present study is comparable to that reported from *S. aureus* surgical site infections from a multicenter Swiss study [26].

Charlson comorbidity index, as expected, was independently associated with a worse outcome [5, 6, 9–11]. Both immunosuppression and septic shock are known to impact mortality of *S. aureus* bacteremia [9, 14, 15]. Since, the vast majority of patients received appropriate empiric antibiotic treatment (90.7%), we could not assess its impact on survival; in previous studies, a smaller percentage of patients received appropriate antimicrobial treatment during the first 24 h (52.8–74.7%), and its administration was associated with better outcome [6, 7, 16].

In accordance to the literature, persistent bacteremia was found to independently predict mortality [5, 27]. In a previous study focusing on duration of persistent bacteremia, mortality increased by 16% for every day of delay in clearance of bacteremia [27]. In that report, delay or absence of source control was associated with persistence of bacteremia [27].

As previously shown, focus of infection plays an important role on outcome, with bacteremias of unknown origin [5, 8, 9, 11] or due to pulmonary infections [8, 11–13] being associated with worse outcomes. In contrast to prior studies, endocarditis was not associated with increased mortality [5, 8, 9]. An explanation for the absence of such association might be the high rates of cardiac surgery (34.3%) among patients with valvular endocarditis in our setting as compared to previous studies (15–26%) [28, 29]. Both previous studies showed that absence of cardiac surgery among patients with endocarditis was associated with worse outcome [28, 29].

The role of early source control on survival among patients with *S. aureus* bacteremia remains debated, since some studies have shown that source control improved survival [6, 7], while in others source control did not confer significant survival benefit [2, 9, 16, 30]. Achieving adequate source control accelerates *S. aureus* bacteremia’s clearance and reduces associated mortality [27]. This essential element of management was not included in the analysis of mortality of many previous studies [8, 10, 11, 14, 15, 31]. There are many confounders for the association of infection source eradication and improved survival, such as decision of care limitation or withdrawal and desistance of surgeon or interventional radiologist to perform the intervention [25]. To account for such a bias, a secondary analysis was performed by including patients that were on maximal care for the first 7 days from infection onset; early source control was associated with better outcome in that subgroup, underlining the importance of prompt control of infection focus on the management of *S. aureus* bacteremia. Our results are in accordance to studies focusing on early source control procedures among other types of infections such us intra-abdominal infections, necrotizing fasciitis and sepsis [32–34]. The timing of source control was different in the present study depending on site of infection and the complexity of each intervention, with catheter removal among patients with unknown origin or catheter-related bacteremia being performed in the majority of patients within 48 h from infection onset, while drainage of abscesses or replacement of prosthetic material being less commonly performed in the same timepoint.

The management of patients with *S. aureus* bacteremia is complex and our results show that infectious diseases consultation within 48 h played an important role in reducing mortality. As it was shown in a previous study from our institution (2001–2010), after the implementation of mandatory infectious diseases consultation for MRSA bacteremia, rates of source control increased leading to improved survival [3]. Infectious diseases consultation was shown to increase adherence to guidelines (follow-up blood culture, echocardiography) and improve management (appropriate antimicrobial treatment, source control) and outcome [12, 31].

The present study has several limitations. First, it is a single center retrospective study, which can make the identification of the source of *S. aureus* bacteremia difficult for some patients. It was conducted in a setting of low MRSA prevalence meaning that results cannot be extrapolated to settings with much higher prevalence. Our cohort represents the most complex cases, since we collected data from patients requiring hospitalization in a tertiary hospital; thus, our epidemiology may not reflect non-tertiary hospitals. Second, no molecular investigation, such exotoxin genes or clonal types, was performed; this area remains scarcely investigated and more research is needed to ascertain the role of superantigens, cytotoxic exotoxins, or various clones on mortality. Additionally, since no clonal types investigation was performed, we could not ascertain if the 60 recurring episodes were from the same or a new *S. aureus* strain.

In conclusion, we found that in patients with *S. aureus* bacteremia delaying source control interventions was associated with worse outcome. Moreover, this study underscores the importance of infectious diseases consultation by guiding antimicrobial treatment, diagnostic investigations and proposing source control interventions.

## Data Availability

The datasets generated during and/or analyzed during the current study are not publicly available, but are available from the corresponding author on reasonable request.
